# Distribution of Platinum and Palladium between Dissolved, Nanoparticulate, and Microparticulate Fractions of Road Dust

**DOI:** 10.3390/molecules27186107

**Published:** 2022-09-19

**Authors:** Mikhail S. Ermolin, Alexandr I. Ivaneev, Anton S. Brzhezinskiy, Natalia N. Fedyunina, Vasily K. Karandashev, Petr S. Fedotov

**Affiliations:** 1 Vernadsky Institute of Geochemistry and Analytical Chemistry, Russian Academy of Sciences, 19 Kosygin Street, 119991 Moscow, Russia; 2 Laboratory of Separation and Pre-Concentration in the Chemical Diagnostics of Functional Materials and Environmental Objects, University of Science and Technology MISIS, 4 Leninsky Prospect, 119049 Moscow, Russia; 3 Institute of Microelectronics Technology and High-Purity Materials, Russian Academy of Sciences, 6 Akademika Osipiana Street, 142432 Chernogolovka, Russia

**Keywords:** platinum, palladium, distribution, dissolved species, nanoparticles, microparticles, fractions, road dust, single particle ICP-MS

## Abstract

Ageing processes of vehicle catalytic converters inevitably lead to the release of Pt and Pd into the environment, road dust being the main sink. Though Pt and Pd are contained in catalytic converters in nanoparticulate metallic form, under environmental conditions, they can be transformed into toxic dissolved species. In the present work, the distribution of Pt and Pd between dissolved, nanoparticulate, and microparticulate fractions of Moscow road dust is assessed. The total concentrations of Pt and Pd in dust vary in the ranges 9–142 ng (mean 35) and 155–456 (mean 235) ng g^−1^, respectively. The nanoparticulate and dissolved species of Pt and Pd in dust were studied using single particle inductively coupled plasma mass spectrometry. The median sizes of nanoparticulate Pt and Pd were 7 and 13 nm, respectively. The nanoparticulate fraction of Pt and Pd in Moscow dust is only about 1.6–1.8%. The average contents of dissolved fraction of Pt and Pd are 10.4% and 4.1%, respectively. The major fractions of Pt and Pd (88–94%) in road dust are associated with microparticles. Although the microparticulate fractions of Pt and Pd are relatively stable, they may become dissolved under changing environmental conditions and, hence, transformed into toxic species.

## 1. Introduction

Platinum group elements (PGEs), such as Pt, Pd, and Rh, are the main active components of the vehicle catalytic converters applied to reduce the emissions of harmful substances (carbon monoxide, nitrogen oxides, hydrocarbons, etc.) from the exhaust system [1,2,3]. During ageing processes, thermal, chemical, and mechanical abrasion of the converters leads to the release of PGEs into the environment [4,5,6,7,8,9]. It is considered that PGEs are released from the converters as particulate forms [2,10]. Though PGEs are used in the catalytic converters as nanoparticulate species (<10 nm) [3], the emitted particles containing PGEs are mainly of micron size range [11]. It is reported that, on average, 66% of emitted PGE-containing particles are greater than 10 μm, 21% of particles are in the range 3–10 μm, and 13% of particles are smaller than 3 μm [11]. PGEs can be major components of the emitted particles and their minor components, for example, if PGEs are present in Al/Si-containing debris of washcoat [12]. The emitted PGE-containing particles are distributed and accumulated in urban environmental compartments. The elevated concentrations of PGE are found in airborne particles [13,14], road dust [13], roadside soil [6,15,16], and aquatic systems [17,18]. The concentrations of Pt and Pd in road dust of different cities are presented in Table 1. As seen from the table, the concentrations of Pt and Pd in urban dust vary significantly from about 1 ng g^−1^ in Stellenbosch (South Africa) to hundreds of ng g^−1^ in Moscow (Russia), London (UK), Perth (Australia), Toronto (Canada), and Chinese cities, such as Hong Kong, Shenzhen, Guangzhou, Beijing. Probably, the difference in concentrations is due to the number of cars and traffic intensity. It should be noted that the ratio of Pt and Pd in dust is also different. For example, in dust of Gothenburg [13], Białystok [19], and Beijing [20], the concentration of Pt is higher than Pd, and vice versa in Rome [13], Moscow [21], Hong Kong, Shenzhen, Guangzhou [22], Beijing [23], and Toronto [24]. This may be due to the different compositions of catalytic converters used in cars in different countries because vehicle emissions are the main source of Pt and Pd in road dust.

The emitted PGEs can present not only as particulate species but also as dissolved ones [2]. In the environment, nanoparticulate species of PGEs can interact with several different ligands and can be transformed into dissolved ones [32], and the latter are more bioavailable [33]. Physiologically based extraction tests show that the bioavailable fraction of PGEs in road dust can reach 68% of their total content, possibly due to the presence of mobile PGE species formed in the roadside environment [34]. It is generally accepted that particulate (metallic) PGEs are nontoxic, while their dissolved species (e.g., chlorinated forms) are toxic and allergenic [35,36]. The toxicity of several PGE-chlorinated salts and evidence of DNA damage have been observed both in vitro and in vivo [35,36,37]. A study on the bioavailability of PGEs in the human digestive tract showed that Pt and Pd did not undergo precipitation reaction when passing from the acid environment of the stomach to the neutral environment of the small intestine [34]. It has also been shown that Pd is more bioavailable than Pt, probably due to the differences in their mobilities and tendencies to form soluble complexes [34]. Besides, the solubilization of PGEs and the formation of chloride complexes are also possible in the human digestive tract, which raises health-hazard issues [34].

It is reported that dissolved Pt is emitted in very small quantities (≤1%) [11]. Other studies show that the dissolved fraction of Pt emitted from fresh gasoline and diesel catalysts is less than 10% [2,38]. The lack of these studies is that “water-soluble” fraction is defined as <0.45 μm, so it comprises both nanoparticulate and ionic fractions. In general, the speciation of PGEs in the environment remains poorly understood [10]. At the same time, the speciation of elements governs their mobility, bioavailability, toxicity, and bioaccumulation. Nanoparticulate metals can be found in solutions as nanoparticles, dissolved ions, and surface-adsorbed ions [39]. Therefore, identifying and/or measuring the quantities of “nanoparticulate elements” in a sample is related to the speciation analysis. The determination and speciation of environmental nanoparticles is an urgent problem of analytical chemistry. The special term “nanospeciation analysis” has already been introduced in studies on the fate of nanoparticles under different conditions [40]. In general, the speciation of elements associated with nanoparticles (nanospeciation) should include two main steps: (1) detection of nanoparticulate elements and (2) determination of chemical species of nanoparticulate elements [41].

The detection of nanoparticulate elements (i.e., the first step of nanospeciation analysis) is possible by using the single particle inductively coupled plasma mass spectrometry (spICP-MS) proposed for the characterization and analysis of colloidal suspensions [42,43,44,45,46]. spICP-MS implies recording time-resolved signals at high acquisition frequencies (10^4^–10^5^ Hz, or dwell time of 10–100 μs). Due to the use of such a fast data acquisition system, detailed information about the transient signal produced by each nanoparticle can be obtained [47]. The basic assumption behind spICP-MS is that each recorded pulse represents a single nanoparticle, while a “steady” signal between the pulses is related to the dissolved species of an element. In turn, the frequency of the pulses is directly related to the number concentration of nanoparticles. The intensity of each pulse is proportional to the mass of the element in each detected nanoparticle [48]. Therefore, spICP-MS can provide important information on environmental nanoparticles, such as size distribution, particle number, particle mass concentration, and concentration of ionic species. Moreover, spICP-MS allows one to determine nanoparticles at the ultratrace level (ng L^−1^), which is very important for the analysis of environment samples.

The aim of the present work is to evaluate the concentration of nanoparticulate and dissolved Pt and Pd in road dust by using spICP-MS. The study is performed taking as example the samples of road dust collected from the Moscow downtown.

## 2. Results and Discussion

### 2.1. Concentration of Pt and Pd in Bulk Road Dust Samples

Pt and/or Pd were found in 22 of 78 road dust samples: in 12 of 28 samples from major highways and in 10 of 42 samples from secondary roads/residential areas. Probably, this is due to the uneven distribution of Pt and Pd in dust samples along the roads. Since preconcentration methods were not used prior to analysis, Pt and Pd were determined only in the samples with concentrations above the limit of detection (LOD). Pt and Pd were not found in dust samples from parks due to the minor effect of traffic emissions. The minimum, maximum, and mean concentrations of Pt and Pd in road dust samples are given in Table 2. It was found that the mean concentrations of Pt and Pd in road dust samples under study were 35 and 235 ng g^−1^, respectively. The determined concentrations are comparable with the concentrations of Pt and Pd in other cities (see Table 1). The concentrations of Pt and Pd in dust found in the present study are more close to the ones found in Hong Kong, Shenzhen, and Guangzhou (China) [22]. Obviously, the concentrations of Pt and Pd in dust are dependent on the intensity of road traffic. For example, in Stellenbosch (South Africa), where traffic intensity is low, the concentrations of Pt and Pd are only about 1 ng g^−1^. As for the ratio of Pt and Pd, the concentration of Pt in Moscow road dust is lower than Pd like in other big cities, such as Rome [13], Hong Kong, Shenzhen, Guangzhou [22], Beijing [23], and Toronto [24].

### 2.2. Particle Size Distribution of Separated Clay Fractions of Road Dust

**Figure 1:**
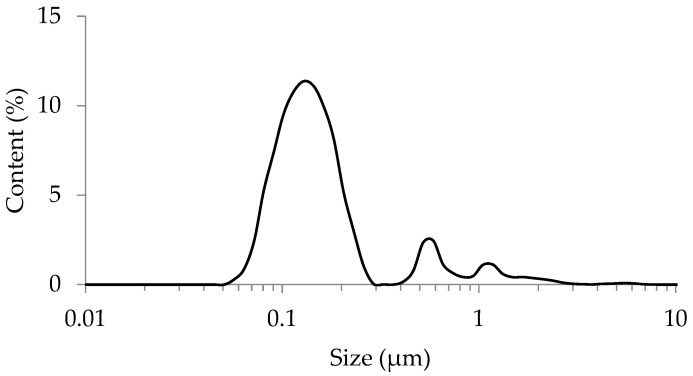
Particle size distribution of a separated clay fraction of road dust.

The clay fractions (<2 μm) of dust samples were separated from the dust samples. The characteristic particle size distribution of dust clay fractions is given in Figure 1. As seen from the figure, the particle size distribution ranges from 50 to 2 μm and has three maxima, 130 nm, 580 nm, and 1.1 μm. It should be noted that a nanosized population (50–300 nm) predominates in a separated clay fraction of dust, which is about 87%. Other two populations in the size ranges 0.4–0.9 μm and 0.9–2 μm account for only about 8% and 5%, respectively. The high content of the nanosized population of particles proves that the separated clay fraction is representative for the determination of nanoparticulate Pt and Pd in road dust by spICP-MS.

### 2.3. Concentration of Nanoparticulate Pt and Pd in Road Dust Samples

For the assessment of the transport efficiency of nanoparticulate matter in spICP-MS measurements, the reference sample of Au nanoparticles was analyzed. The results are presented in Table 3. It has been shown that the determined experimental values of size, number concentration, and mass concentration of Au nanoparticles are in good agreement with the certified values. A slight deviation of experimental results from the specification can be attributed to inevitable inaccuracies of multiple dilutions.

**Figure 2:**
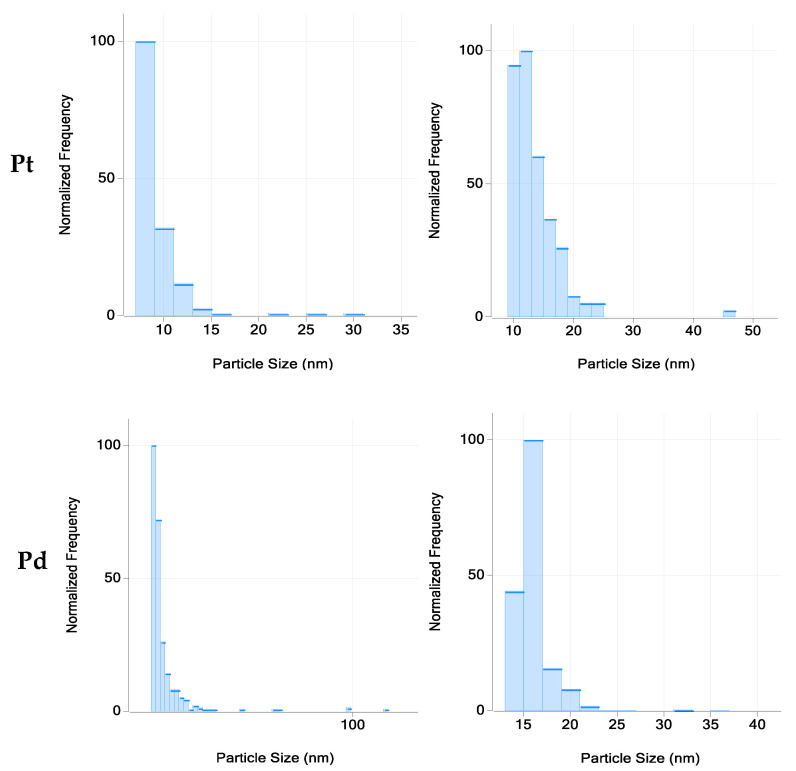
Typical examples of particle size distribution of nanoparticulate Pt and Pd as obtained by spICP-MS.

The results of the spICP-MS analysis of clay fractions of road dust samples are presented in Table 4. It is shown that road dust contains nanoparticles of Pt and Pd with sizes in the ranges 6–11 nm and 10–21 nm, respectively. Typical examples of particle size distributions of nanoparticulate Pt and Pd are given in Figure 2. It should be noted that Pt nanoparticles with a comparable size (8–21 nm) were earlier identified in road dust of Ghent and Gothenburg [10]. The mean concentration of nanoparticulate Pt and Pd in the separated clay fractions of dust are 1.6 and 21 ng L^−1^, respectively. spICP-MS analysis also enables ionic concentrations of elements to be determined. It has been found that the mean concentrations of dissolved species of Pt and Pd in the separated clay fractions of road dust are 12 and 90 ng L^−1^, respectively. It is revealed that the concentrations of dissolved species of Pt and Pd are about 4–10 times higher than the concentrations of their nanoparticulate species. This can be explained by the gradual dissolution of metallic Pt and Pd in the urban environment. The dissolution of particulate Pt and Pd in the urban environment can occur, for example, as a result of rains and/or watering (washing) the roads. It should be noted that the latter is a common procedure in Moscow in the summer period.

The concentrations obtained by spICP-MS were recalculated into the concentrations of nanoparticulate and dissolved species of Pt and Pd in bulk dust samples. The concentrations of nanoparticulate species of Pt and Pd in Moscow road dust varied in the ranges <LOD–2.7 (mean 0.3) ng g^−1^ and 0.4–15.6 (mean 3.1) ng g^−1^, respectively. The concentrations of dissolved species of Pt and Pd were 0.2–5.1 (mean 1.7) ng g^−1^ and 2–106 (mean 14) ng g^−1^, respectively.

### 2.4. Association of Pt and Pd with Nanoparticulate, Microparticulate, and Dissolved Fractions of Road Dust

**Figure 3:**
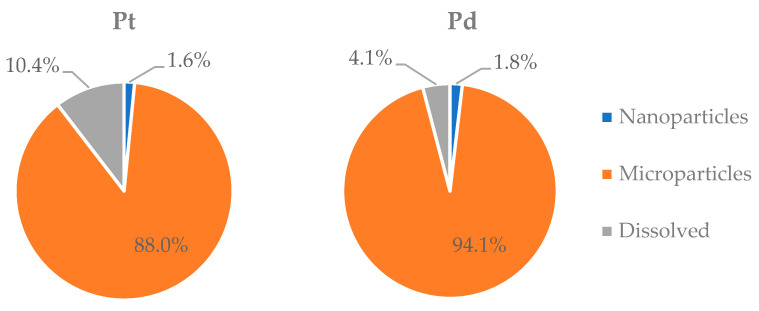
Distribution of Pt and Pd between nanoparticulate, microparticulate, and dissolved fractions of road dust.

Based on the results of the analysis of bulk (<100 μm) road dust samples and spICP-MS analysis, the ratio of nanoparticulate, microparticulate, and dissolved fractions of Pt and Pd in road dust was evaluated (Figure 3). According to the IUPAC definitions, microparticles have sizes from 0.1 to 100 μm, while nanoparticles have sizes from 1 to 100 nm [49]. It has been revealed that the nanoparticulate fraction of Pt and Pd in Moscow road dust is only about 1.6–1.8%. The content of a dissolved fraction of Pt and Pd varies in the range 4–10%. Earlier [10], it was found that, on average, 3.3% of Pt in road dust is in nanoparticulate form; this is comparable with the concentrations determined in the present study. In contrast, no dissolved Pt was found in the abovementioned work [10]. It is found that the average content of dissolved fraction is higher for Pt than for Pd. The representative and well-studied species of Pt and Pd in natural waters are neutral or slightly negatively charged hydroxy- or hydroxochloride complexes [50]. Platinum (IV) hydroxide (Pt(OH)_4_⋅mH_2_O) has higher solubility as compared with Pd species (aquated and hydrolyzed chloride complexes) [50]. The solubility of the hydroxocomplexes of PGEs varies in the following order: Pt > Rh > Pd [50]. This can be the reason for the higher content of dissolved fraction of Pt as compared with Pd.

It is known that nanoparticles are metastable and can dissolve in aqueous systems; besides, sonication can also intensify the process of dissolution. Therefore, the sample preparation process (10 min of sonication) may contribute to the dissolved fraction of Pt and Pd found in the present study.

The largest amounts of Pt and Pd in Moscow road dust are associated with the fraction of microparticles (88–94%). Probably, this can be explained by the presence in dust micron-sized washcoat debris containing PGE nanocoating. Though the microparticulate fraction of dust does not have such mobility in the environment as nanoparticles, it can contribute to dissolved fractions of Pt and Pd by gradual dissolution. On the other hand, due to aggregation, nanoparticles of Pt and Pd can also be immobilized on the surface of dust microparticles. As a result, the recovery of nanoparticulate Pt and Pd may be underestimated.

## 3. Materials and Methods

### 3.1. Road Dust Sampling

**Figure 4:**
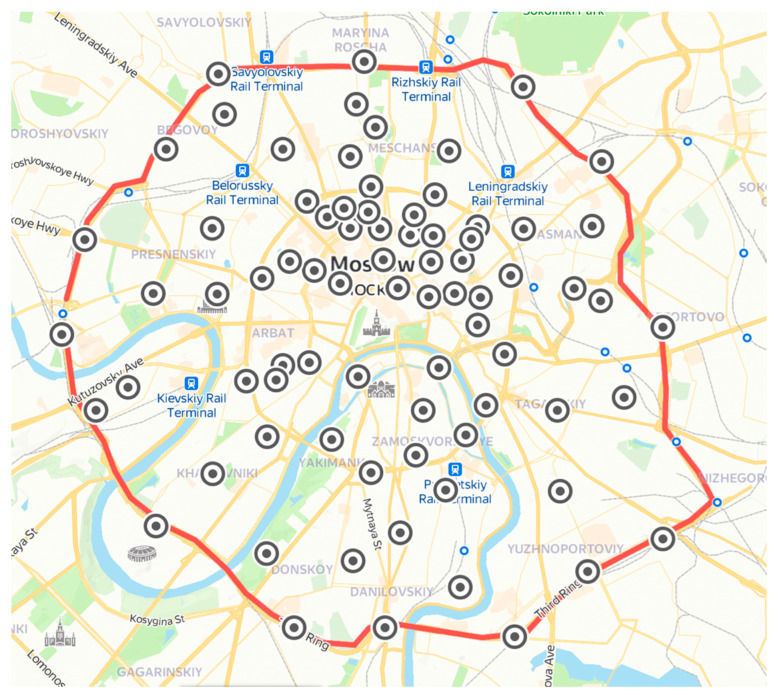
Map of the Moscow downtown with indicated dust sampling sites. Red line indicates the Third Transport Ring.

The sampling of dust was carried out in the Moscow downtown (city center) on the territory bounded by a major highway, the Third Transport Ring. Dust samples were collected from major roads (28 samples), secondary roads (11 samples), parks (7 samples), and residential areas (32 samples); a total of 78 dust samples were collected (Figure 4). It should be noted that residential areas are surrounded by the secondary road, so the difference between these sampling areas is debatable. The sampling was carried out on 10–14 July 2021 under dry weather conditions. For 2 weeks prior to the start of the sampling, no precipitation was observed, the air temperature was extremely high and reached a record of 34 °C, atmospheric pressure averaged at 750 mm Hg, atmospheric air humidity varied from 40% to 75%, and the wind was predominantly south, southwest with an average speed of 1 m s^−1^ [51]. The dust samples were carefully collected with a polypropylene brush to avoid a resuspension of fine dust particles in the air as much as possible. As a rule, dust nanoparticles are attached to the surface of larger (micron-sized) particles of dust, so we assume that the loss of nanoparticles during dust sampling is minimum. Then, the samples were weighed and sieved through 100 μm to remove coarse particles and debris. The average weight of each collected dust sample was 124 ± 44 g, whereas after sieving, it was 18 ± 9 g, which was, on average, 14% of the initial weight of the samples collected.

### 3.2. Determination of Platinum and Palladium in Road Dust Samples

The determination of Pt and Pd in dust samples was performed using ICP-MS after acid digestion. The dust samples (100 mg) were digested in an open beaker by using four acids (HF, HNO_3_, HClO_4_, and HCl). The procedure of total digestion is described in detail earlier [52].

For the determination of Pt and Pd, an XSeries mass spectrometer (Thermo Scientific, Waltham, MA, USA) was used with standard settings: generator output power of 1250 W, concentric PolyCon nebulizer, quartz cooled spray chamber (3 °C), plasma argon flow rate of 13 L min^−1^, flow rate of the auxiliary argon flow of 0.9 L min^−1^, flow rate of argon in the nebulizer of 0.89 L min^−1^, flow rate of the analyzed sample of 0.8 mL min^−1^, and resolution 0.8 M. The limit of detection was calculated as LOD = 3 s, where s is the standard deviation in the analysis of control samples.

To control the correctness of the analysis of dust samples, the reference samples were AMIS0192 (certified reference material, platinum (PGM), Merensky ore, Bushveld Complex, South Africa), AMIS0395 (certified reference material, platinum (PGM), Platreef ore, Bushveld Complex, South Africa), and DGPM-1 (US Geological Survey, Pinson Mine disseminated gold).

### 3.3. Separation of Clay Fractions of Road Dust Samples

Before spICP-MS analysis, the clay fractions (<2 μm) of dust samples were separated by centrifugation. For that, 15 mL of suspensions containing 100 mg of dust in deionized water were prepared. Then, for the mobilization of nanoparticles, the suspensions were treated in the ultrasound bath (Bandelin Sonorex) for 10 min. After that, the suspensions were centrifuged for 1 min at 3500 rpm. The centrifugation parameters (time and rotation speed) were calculated for the sedimentation of particles >2 μm. The particle size distributions of separated clay fractions were controlled by laser diffraction (Shimadzu SALD-7500nano). The supernatants (clay fractions) were decanted and divided into two portions. One portion was filtered through 20 kD to remove particles from the solutions and to obtain a dissolved fraction. The dissolved fractions were used as control samples for spICP-MS analysis. Both control samples and clay fractions were analyzed by spICP-MS.

### 3.4. SpICP-MS Determination of Pt and Pd in the Clay Fraction of Road Dust

The set of clay fractions of dust and control samples (filtrates of clay fractions) was analyzed using Agilent 7900 ICP-MS in the single particle analysis mode. The separated clay fractions and control samples were not diluted before the analysis. The following parameters of ICP-MS were used: a RF generator power of 1550 W, a Peltier-cooled Scott spray chamber (2 °C), a MicroMist nebulizer, a plasma-forming Ar flow rate of 15 L min^−1^, an Ar flow rate into the nebulizer of 0.90 L min^−1^, an analyzed sample flow rate of 1.0 mL min^−1^, an auxiliary gas flow rate of 1 L min^−1^, and a dwell time of 0.1 ms. A quartz torch with a 1.5 mm injector was used in single particle detection mode. The standard solutions (high-purity standards) were used for calibration. The analysis was performed in multielement SP mode without settling time. Elements were determined sequentially for 20 s each. The following *m/z* values were monitored: ^195^Pt and ^105^Pd. The transport efficiency was determined using the particle size method by using reference nanoparticles of known size [53], namely, the sample of 30 nm ultrauniform gold nanospheres in 2 mM sodium citrate solution (0.053 mg mL^−1^, 2.1 × 10^11^ particles mL^−1^ nanoComposix). For the determination of the transport efficiency, the suspension of Au nanoparticles with a concentration of 53 ng L^−1^ in 2 mM sodium citrate solution was prepared and analyzed by spICP-MS. The results of the analysis were processed by an Agilent 7900 software (MassHunter 4.4). Peak integration mode was used for processing the results. The control samples were used for the determination of baseline and threshold values. 

The control samples (filtrates of clay fractions) were used for the correct determination of particle baseline and particle detection threshold. The signal of nanoparticles leads to the overestimation of the determined particle baseline and particle detection threshold and, hence, to the underestimation of nanoparticle concentration. Therefore, the values of the particle baseline and particle detection threshold (calculated as 6s, with s-standard deviation) obtained for the control samples were used for processing the results for the suspensions (clay fractions).

## 4. Conclusions

Single particle inductively coupled mass spectrometry has been proven to be a powerful tool for the determination and characterization of nanoparticulate Pt and Pd in environmental samples. For road dust of Moscow, it has been found that nanoparticulate and dissolved fractions of the studied elements are only about 1.6–1.8% and 4–10%, respectively. The major portion of Pt and Pd (88–94% of the total content) is associated with the microparticulate fraction of Moscow road dust. Despite the low mobility, the microparticulate fractions of Pt and Pd can release potentially toxic dissolved species as a result of gradual dissolution. Therefore, the processes of dissolution and the environmental conditions, under which the dissolution occurs, require further investigation.

## Figures and Tables

**Table 1:** The abundance of Pt and Pd in road dust.

Concentration, ng g^−1^		Location	Reference
Pt	Pd		
317	-	Madrid, Spain	[25]
326 58 74 34 173	71 - - 203 -	Gothenburg, Sweden Sheffield, UK London, UK Rome, Italy Munich, Germany	[13]
0.16–1.28	0.64–1.76	Stellenbosch, South Africa	[26]
5–79	-	Ghent, Belgium Gothenburg, Sweden	[10]
28	58	Beijing, China	[23]
12–357 (mean 71)	8–225 (mean 158)	Moscow, Russia	[21]
3.8–444 (mean 115)	-	Seoul, Korea	[27]
34–111	33–42	Białystok, Poland	[19]
1.5–43	1.2–58	Hyderabad, India	[28]
12–187 14–178 5–48	12–287 34–514 13–554	Hong Kong, China Shenzhen, China Guangzhou, China	[22]
35–131	10–88	Houston, USA	[29]
4–356 (mean 97)	0.1–125 (mean 20)	Beijing, China	[20]
102–764	-	London, UK	[30]
54–419	58–440	Perth, Australia	[31]
<5–151	10–516	Toronto, Canada	[24]

**Table 2:** Concentrations of Pt and Pd in road dust as obtained by ICP-MS.

Element	Concentration, ng g^−1^			
	LOD	Mean	Minimum	Maximum
**Pt**	7	35	9	142
**Pd**	50	235	155	456

**Table 3:** Particle size, particle number, and mass concentration for the reference sample of Au nanoparticles (*n* = 3).

	Size (nm)	Number Concentration (Particles L^−1^)	Mass Concentration (ng L^−1^)
**Certified values**	29.4 ± 1.3	2.1 × 10^8^	53
**Experimental results**	29.2 ± 0.1	(2.0 ± 0.3) ×10^8^	54 ± 8

**Table 4:** Concentrations of nanoparticulate and dissolved species of Pt and Pd in clay fractions of road dust as obtained by spICP-MS.

	Particle Concentration (Particles L^−1^)	Mass Concentration (ng L^−1^)	Ionic Concentration (ng L^−1^)	Median Size (nm)	Size Detection Limit (nm)
**Pt**					
**Mean**	2.8 × 10^8^	1.6	12	7	3.5
**Minimum**	1.2 × 10^6^	0.03	1	6	2.8
**Maximum**	3.4 × 10^9^	18.2	34	11	5.1
**Pd**					
**Mean**	1.1 × 10^9^	21	90	13	6.7
**Minimum**	5.7 × 10^7^	3	13	10	4.4
**Maximum**	3.0 × 10^9^	104	708	21	13.9

## Data Availability

Not applicable.
